# Early operable breast cancer in elderly women treated with an aromatase inhibitor letrozole as sole therapy

**DOI:** 10.1038/bjc.2011.470

**Published:** 2011-11-08

**Authors:** A Balakrishnan, D Ravichandran

**Affiliations:** 1 Breast Unit, Luton & Dunstable Hospital NHS Foundation Trust, Lewsey Road, Bedfordshire, Luton LU4 0DZ, UK

**Keywords:** breast cancer, aromatase inhibitors, letrozole

## Abstract

**Background:**

Primary endocrine therapy (PET) with aromatase inhibitors (AIs) is an option in elderly patients unfit for or unwilling to undergo surgery. We studied the outcome of patients treated with letrozole as PET.

**Methods:**

Patients with early oestrogen receptor (ER)/progesterone receptor (PR)-positive breast cancer treated with letrozole from February 2001 to September 2009 were reviewed. Inoperable and locally advanced tumours were excluded. Reasons for offering PET, response, survival, cause of death, time to initial and best response, fracture incidence, and late failure rates were studied.

**Results:**

In all, 104 patients received PET due to frailty (*n*=48), comorbidity (*n*=30), old age (*n*=9), and patient preference (*n*=17). Median follow-up was 56 months (4–106). Eighty-five cancers responded to letrozole (stable disease (SD, *n*=19), reduction in size (PR, *n*=42), and complete response ((CR), *n*=24)). Median survival was 51 months (4–103), time to initial response (PR/CR) 4.5 months (2–24), and time to best response 8.5 months (3–50). Letrozole was stopped in 25 patients due to progressive disease (*n*=19), side effects (*n*=5), and patient choice (*n*=1). Only 12 of 49 deaths were from breast cancer.

**Conclusion:**

Letrozole is a reasonable alternative in elderly women with early ER/PR-positive invasive breast cancer who are unfit or unwilling to undergo standard therapy.

Female sex and increasing age are by far the strongest risk factors for breast cancer and the increase in life expectancy has resulted in a greater number of elderly women presenting with breast cancer. Surgery, followed by appropriate adjuvant therapy, remains the optimal management for women with early breast cancer regardless of age. However, a significant proportion of elderly patients presenting with breast cancer is either unfit for surgery due to comorbidity, or less commonly, unwilling to undergo operative intervention.

Endocrine therapy has long been employed as adjuvant therapy after surgery in those with oestrogen receptor (ER)- and/or progesterone receptor (PR)-positive tumours and is very effective in reducing recurrences and death (Early_Breast_Cancer_Trialists’_Collaborative_Group, 2001, 2005; Baum *et al*, 2002). Endocrine therapy is also successful as neoadjuvant therapy, allowing surgery in those presenting with irresectable tumours and allowing breast conservation in those presenting with large tumours (Dixon *et al*, 2000, 2001; Eiermann *et al*, 2001; Mauriac *et al*, 2002; Ellis *et al*, 2011). Primary endocrine therapy (PET), where the cancer is treated solely with an endocrine agent, is an option in women with an ER/PR-positive tumour who are unsuitable or unwilling to undergo surgery (Milla-Santos *et al*, 2004; Crivellari *et al*, 2008; Muss *et al*, 2008). Twenty-six percent of women aged 70–79 years and 61% of women aged 80 years and above diagnosed with breast cancer in the UK have no surgical treatment recorded and it is likely that most of these patients are treated with PET (Traa *et al*, 2011). Tamoxifen has been the PET drug of choice for a number of years (Crivellari *et al*, 2008). Over the last 10–15 years, aromatase inhibitors (AIs) have been shown to be better than tamoxifen in multiple clinical scenarios in breast cancer including the neoadjuvant setting (Dixon *et al*, 2000, 2001; Nabholtz *et al*, 2000; Eiermann *et al*, 2001; Mouridsen *et al*, 2001; Baum *et al*, 2002; Thurlimann *et al*, 2005; Crivellari *et al*, 2008; Ellis *et al*, 2011). Based on these results, AIs are also replacing tamoxifen as PET (Mathew *et al*, 2009) but there have been few published studies on the role of these drugs as PET especially in women with early operable breast cancer. We reviewed our experience of using letrozole, an AI, as PET in these women.

## Materials and methods

We identified all patients who received letrozole as sole therapy after presenting with primary breast carcinoma to one breast unit over a period of eight and a half years between February 2001 and September 2009. These patients were identified from a prospectively entered electronic database from the year 2005 and from breast cancer multidisciplinary meeting minutes for the earlier period. Both symptomatic and screen-detected breast cancers were included. The entry criteria were invasive cancer confirmed histologically by core biopsy, ER and/or PR positivity with no evidence of metastatic, locally advanced, or inoperable tumours and no planned surgery at a later stage. The ER/PR positivity was determined in the early part of the study by >30% tumour cell nuclei staining positive with immunostaining for ER and/or PR and in the later part of the study by an Allred Score of 4 or more. Definition of locally advanced/inoperable tumours was any tumour that may not be safely and completely removed by a standard mastectomy based on a clinical assessment by a consultant breast surgeon. Imaging for metastatic disease was only performed when there is a clinical suspicion of metastatic disease. Thus, all patients had surgically resectable breast carcinoma. These patients made the study population and their case notes were reviewed. The date of censoring for data collection was December 2009.

The primary end point was survival and cause of death. Secondary end points were response rate, time taken to achieve a tumour response, time taken to achieve the best response seen with letrozole, failure rate after an initial response and the incidence of bone fractures, the main adverse effect of AIs. Assessment of tumour response was mainly by clinical examination; a complete response (CR) was defined as a tumour that was initially palpable but became impalpable on treatment with letrozole, while a partial response (PR) was defined as a palpable tumour that had decreased in size. Imaging assessment was done very infrequently in these patients. Reasons for offering PET rather than surgery were recorded in addition to tumour size and histology, axillary nodal status, and the number of follow-up visits. Frequency of follow-up visits after commencing on letrozole varied depending on patients’ condition (e.g., age and mobility) and clinician's concern. In general, patients were seen more frequently at the commencement of therapy to identify the non-responders and when there was a concern that tumour might be escaping control.

## Results

### Patient demographics, tumour size, and nodal status

A total of 104 female patients fulfilled the entry criteria. Their age, mode of presentation, the histological type of cancer, tumour size, the reasons for using PET (rather than surgery), and follow-up details are given in Table 1. Median follow-up period (defined as the time from diagnosis to the date of death or censoring, *n*=104) was 56 months (4–106). Axillary nodes were clinically palpable in 22 patients at the time of presentation. This study took place before the introduction of routine axillary nodal fine needle aspirate to our practice in the presence of ultrasonically suspicious axillary nodes. Thus, only 3 patients out of 104 had cytological confirmation of a positive axilla.

### Survival

**Figure 1:**
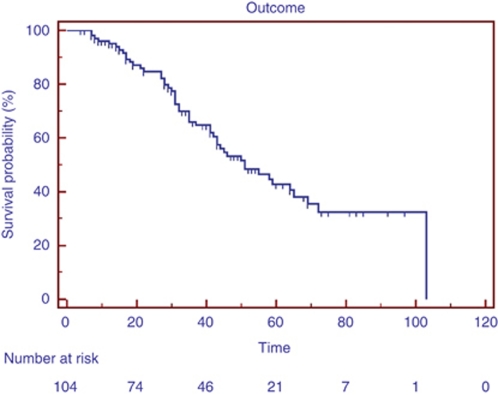
Kaplan–Meier survival plot for all patients (*n*=104).

Median survival was 51 months (4–103, Figure 1). Fifty-five patients were alive at the time of censoring and thirty-eight were still on letrozole. Forty-nine patients had died by the time of censoring. Cause of death was available in 42 of these patients; 12 died from breast cancer and 30 from other causes. Cause of death was unavailable in seven, among whom the last known tumour status before death was CR in three, PR in one, progressive disease in one, and unknown in two.

### Tumour response

Overall, 85 of the 104 patients (82%) responded to letrozole and the best response seen was stable disease (SD) in 19 patients, PR in 42, and CR in 24. The median time to show an initial response (PR or CR) was 4.5 months (2–24). The median time to achieve the best response seen with letrozole was 8.5 months (3–50). In all, 60 of 85 patients who responded remained on letrozole as sole therapy until their death or the date of censoring. Letrozole was stopped in others (*n*=25) due to progression of the disease following an initial response (*n*=19), side effects (*n*=5), and patient choice in one patient who opted for surgery. Of the 19 patients with progressive disease, 5 went on to have surgery, 13 were prescribed an alternative AI, and 3 were prescribed tamoxifen. Those patients in whom letrozole was stopped due to side effects were prescribed another AI (*n*=3) or tamoxifen (*n*=2).

### Fracture incidence

Twelve patients sustained fractures during the study period. Two were pathological fractures of spine due to bone metastases. Among others, the duration of letrozole therapy before the fracture varied from 6 to 60 months (mean 31 months) and the commonest fractures were that of femur (*n*=5) and wrist (*n*=3). Eighty-four patients did not sustain a fracture during the follow-up period and fracture data were not available in eight patients.

## Discussion

This appears to be one of the very few published studies looking at the outcome of breast cancer patients treated with an AI as sole therapy, and consists only of patients with surgically resectable disease who were recruited over a relatively short time period and with reasonable follow-up. The retrospective nature of the study introduces a few limitations; the most significant in our opinion is the lack of assessment of comorbidity and expected survival at presentation. Elderly breast cancer patients often present with a number of other comorbidities at diagnosis which influence survival (Satariano and Ragland, 1994). It is impossible to assess comorbidity at presentation accurately in a retrospective study so we did not attempt this. In addition, some patients treated with letrozole before the introduction of an electronic database at our institution in 2005 may have been missed from inclusion in the study and there was inconsistency in follow-up between the patients. Furthermore, tumour response to letrozole in our study was only assessed clinically. Ultrasound and mammograms may have contributed to the assessment process but, like clinical examination, are not exempt from intra- and inter-observer variation. Indeed several published randomised studies on neoadjuvant endocrine therapy have relied on assessment of tumour size by clinical palpation as the main outcome measure (Eiermann *et al*, 2001; Baselga *et al*, 2009; Ellis *et al*, 2011). The main strength of our study is that all cancers were confirmed histologically by core biopsy, only ER/PR-positive patients were included and all patients received letrozole as the only treatment for their breast carcinoma.

Breast cancer is common in the elderly (Lilienfeld, 1963; Louwman *et al*, 2008; NHS_Cancer_Screening_Programmes); 31% of the 50 286 cancers diagnosed in the UK in 2007 were in those aged 70 years or over (Traa *et al*, 2011). A significant proportion of very elderly women are either unfit or unwilling to undergo standard therapy, despite the low morbidity associated with breast cancer surgery. The proportion of ER-rich tumours is greater in the elderly (Cheung *et al*, 2008), rendering PET as an alternative, minimal-risk treatment modality that is acceptable to these women. It would appear that up to 44% of elderly women in the UK may be treated with PET (NHS_Cancer_Screening_Programmes) and 32% of elderly women in two European studies received PET (Bouchardy *et al*, 2003). Primary endocrine therapy was first described in 1982 using tamoxifen (Preece *et al*, 1982) and this was followed by a number of published studies about its effectiveness. A Cochrane review of seven randomised trials comparing tamoxifen only with surgery with or without adjuvant tamoxifen showed PET with tamoxifen was associated with inferior local disease control but similar overall survival (Hind *et al*, 2007), with only one out of seven trials showing a survival advantage for surgery (when combined with adjuvant endocrine therapy) after 13 years of follow-up. It should be noted that only one of these trials recruited women with only ER-positive tumours and the ER status of all other trial participants was unknown. As tamoxifen is unlikely to have been effective in ER-negative tumours this would have placed the tamoxifen arm at a disadvantage. Similarly, quality of surgery, assessment of surgical margins, and the use of adjuvant radiotherapy in some trials were suboptimal by present day standards, reducing the benefit of surgery.

Aromatase inhibitors have shown to be more effective than tamoxifen in various scenarios in breast cancer, generally with a lower toxicity profile (Eiermann *et al*, 2001; Crivellari *et al*, 2008; Cuzick *et al*, 2010). These include metastatic or advanced disease (Riemsma *et al*, 2010) and as adjuvant therapy after surgery (Mouridsen *et al*, 2009; Cuzick *et al*, 2010) in early breast cancer. They are also effective as extended adjuvant therapy after 5 years of tamoxifen in elderly women with node-positive disease (Muss *et al*, 2008). All three third-generation AIs (anastrozole, exemestane, and letrozole) have also been shown to be effective in the neoadjuvant setting, which is relevant to PET (Milla-Santos *et al*, 2004; Mustacchi *et al*, 2009; Olson *et al*, 2009; Toi *et al*, 2011). They have been compared with tamoxifen in this setting. Two randomised trials comparing 3 months of anastrozole with tamoxifen have shown no difference in objective tumour response rate although breast conservation rates were higher in the anastrozole group (Smith *et al*, 2005; Cataliotti *et al*, 2006). It is likely that the treatment duration was not adequate in these trials. Four months of letrozole resulted in significantly better tumour response rates and breast conserving surgery rates compared with tamoxifen in another randomised study (Eiermann *et al*, 2001). Similar results were reported with exemestane, another AI, confirming its superiority over tamoxifen in the neoadjuvant setting (Semiglazov *et al*, 2005), but a small randomised trial has suggested that it may be inferior to letrozole (Chow *et al*, 2008). A recent randomised neoadjuvant trial compared the three AIs in patients with stage 2 and 3 disease (Ellis *et al*, 2011) in which treatment was given for 16–18 weeks. Letrozole was associated with the highest clinical response rate. These results suggest letrozole might be the best agent to be used as PET. Its superior efficacy might be due to better inhibition of whole-body aromatisation and more complete suppression of plasma and intra-tumour oestrogen levels (Geisler, 2011).

Published studies of PET using AIs in those with surgically resectable disease are rare. In a retrospective study of 64 women aged over 70 years who had surgically resectable disease but were either unfit or declined surgery and treated with anastrozole, an objective response rate of 40% and progression-free survival of 16.5 months (2–78) were seen (Okunade *et al*, 2009). Our study demonstrated an objective response rate of 63.5% with letrozole but it should be noted that our criteria for PR was any reduction in tumour size as opposed to WHO criteria of a reduction of ⩾50%. The clinical response rates (combined CR and PR) for letrozole vary from 55% in randomised trials (Eiermann *et al*, 2001) to 62% in non-randomised neoadjuvant trials (Olson *et al*, 2009). Treatment durations used in these trials were generally 16–18 weeks. Our results show that a similar length (4.5 months) of treatment is needed to see a convincing initial response in the form of tumour size reduction although a much shorter time (66 days) has been reported by a neoadjuvant trial (Eiermann *et al*, 2001). The reason for this difference is not clear but the median age of patients in this trial was much lower than ours (68 *vs* 83). It should be noted that the time required for letrozole (2.5 mg once daily) to reach steady-state plasma concentrations has been reported to be 2 months although maximal oestrogen suppression seems to occur within 2–4 days (Buzdar *et al*, 2002).

We observed that median treatment duration of 8.5 months was necessary to achieve the best response produced by letrozole. The observation that longer duration of letrozole therapy results in further tumour volume reduction was also made by Dixon *et al* (2009) and another smaller study (Krainick-Strobel *et al*, 2008). In the study by Dixon *et al* (2009), 182 patients were commenced on neoadjuvant letrozole but about a third of patients (*n*=63, median age 79 years) continued to take the medication beyond the planned 3-month period with response rates increasing with time. Their study included patients with inoperable locally advanced disease. In a phase 2 multicentre study of 117 patients (median age 80 years), 6 months of neoadjuvant exemestane produced a clinical response rate of 70% but the best response was seen only in 33% of patients by 3 months of therapy (Mustacchi *et al*, 2009). These results support our observation that a much longer period of treatment than the standard 16–18 weeks studied in neoadjuvant trials is necessary to achieve the best tumour response seen with AIs.

Eighteen percent of the tumours in our study progressed on letrozole despite being ER/PR positive, which is higher than the 2.2–12% reported in the neoadjuvant letrozole trials (Eiermann *et al*, 2001; Olson *et al*, 2009). In addition, a significant proportion of tumours that initially responded (22% in this study) subsequently escaped control, thereby necessitating other modalities of treatment such as surgery at a later stage and at a more advanced age. It is obvious that we need more reliable markers than just ER and PR to predict response to endocrine therapy. Neoadjuvant AI therapy is associated with profound changes in gene expression and biochemical profiling of the relevant transcripts either at initiation of therapy, or more realistically, after a relatively short duration of treatment (by repeat core biopsy), may accurately predict tumour behaviour and allow early salvage of those which are likely to progress (Yamashita *et al*, 2009; Mello-Grand *et al*, 2010). The challenge is to identify molecular and proliferative changes that would correlate well with clinical and pathological response (Miller and Larionov, 2010; Miller *et al*, 2010). Until this is achieved in everyday clinical practice, close observation of patients is necessary in the initial period to identify those who progress on PET.

Letrozole, by significant inhibition of production of oestrogen can cause bone loss and increased incidence of fractures, its main adverse effect in the elderly as shown in the large adjuvant letrozole trial BIG 1–98 (Crivellari *et al*, 2008). The fracture rate in our study after median follow-up of 56 months was 12.5% compared with 9.3% in the BIG 1–98 trial after a median follow-up of just over 60 months (Rabaglio *et al*, 2009), with fracture risk increasing with age. In both studies, no specific or consistent efforts were made to monitor bone density and no calcium/vitamin D supplements or bisphosphonates were actively recommended. Only 6% (*n*=146) of the BIG 1–98 trial population was ‘elderly’ (⩾75) and the fracture rate in this group was 11.6% after a median follow-up of 40 months (Crivellari *et al*, 2008), in line with our findings. These results suggest that older patients who are prescribed letrozole as PET would benefit from bone density monitoring and prophylactic treatment of those at risk of a fracture, as the risk is similar to those receiving letrozole as adjuvant treatment.

In summary, this study suggests that letrozole is a reasonable alternative in elderly women with ER/PR-positive invasive breast cancer who are either unfit with limited life expectancy or decline standard therapy. Despite the relative richness of ER in this population close observation is necessary as ∼1 in 5 patients may not respond to letrozole and one fifth of those who initially respond may progress after a period of time. These two features make PET, even with an AI, decidedly inferior to surgery. A reduction in tumour size may take on average 4–5 months to manifest and it may take twice as long to see the best tumour response that may be achieved with letrozole. Although the mortality is high in this population, most deaths are due to comorbidity or age rather than breast cancer.

## Figures and Tables

**Table 1:** Patient and tumour characteristics

Number of patients (total)	104
Age (years)	83 (58–98)
Presentation	Symptomatic (*n*=99)
	Screening (*n*=5)
Core biopsy histology	Invasive ductal (*n*=79)
	Invasive lobular (*n*=14)
	Mucinous (*n*=7)
	Colloid (*n*=2)
	Mixed (*n*=2)
Tumour size (mm)	30 (10–100)
	
*ER status*	
>30% tumour cell nuclei staining positive	*n*=36
Allred score ⩾4	*n*=68, median score 8 (4–8)
	
*PR status*	
>30% Tumour cell nuclei staining positive	*n*=25
<30% Tumour cell nuclei staining positive	*n*=8
Allred score ⩾4	*n*=51, median score 5 (0–8)
PR not done	*n*=20
	
Reasons for primary endocrine therapy	Frailty (*n*=48)
	Comorbidity (*n*=30)
	Patient preference (*n*=17)
	Old age (*n*=9)
Follow-up in months (range)	56 (4–106)
Number of follow-up visits per patient	6 (1–20)

Abbreviations: ER=oestrogen receptor; PR=progesterone receptor.

Where applicable, data are displayed as median with the range given in brackets.
